# Man vs. machine: surgeon vs. elastography assessment of the quality of the rotator cuff

**DOI:** 10.1016/j.jseint.2023.02.005

**Published:** 2023-03-05

**Authors:** Alexander G. Maloof, Lisa Hackett, Patrick H. Lam, George A.C. Murrell

**Affiliations:** a Orthopaedic Research Institute, St George Hospital, Sydney, New South Wales, Australia; b University of New South Wales, Sydney, New South Wales, Australia

**Keywords:** Rotator cuff repair, Shear wave elastography, Supraspinatus tendon stiffness, Surgeon rankings, Tendon quality, Man versus machine, Surgeon vs ultrasound, Supraspinatus quality

## Abstract

**Background:**

The most common complication of arthroscopic rotator cuff repair is retear, which is more common in larger tears and older patients. We hypothesized that the quality of the torn tendon is important in protecting against retear. Surgeons have traditionally assessed the quality of repaired tendons with a four-point Likert scale. Shear Wave Elastography Ultrasound (SWEUS) is a recent technological advancement that can quantify soft-tissue stiffness. This study aimed to determine how closely a surgeon’s intraoperative ranking of tissue quality during rotator cuff repair correlated to postoperative supraspinatus tendon stiffness measured by SWEUS.

**Methods:**

This was a prospective case series on 50 patients undergoing arthroscopic rotator cuff repair, involving SWEUS measurements of each patient’s supraspinatus tendon at 8 days, 6 weeks, 12 weeks, 6 months, and 12 months. The intraoperative surgeon score of tissue quality for each patient was ranked on a four-point Likert scale. Each patient’s scores were compared to postoperative SWEUS velocity measurements of the supraspinatus tendon postrepair.

**Results:**

The SWEUS determined stiffness of supraspinatus tendons at their repaired insertion site postrepair increased by 22% from 6.3 ± 0.2 m/s to 7.7 ± 0.3 m/s over 12 months as the tendons healed (*P* = .0001). Supraspinatus tendon stiffness was greater in patients with smaller tears (*r* = −0.50, *P* = .001) and of younger age (*r* = −0.58, *P* = .00001). Surgeons also consistently rated younger patients (*r*_*s*_ = −0.49, *P* = .0001) and smaller tears (*r*_*s*_ = −0.56, *P* = .00001) as having superior intraoperative tendon quality. The correlations between SWEUS velocity and surgeon tissue quality rankings were modest at best and strongest at 12 weeks (*r*_*s*_ = 0.27, *P* = .04). There were modest associations between SWEUS tendon stiffness and surgeon tendon mobility rankings at 6 weeks (*r*_*s*_ = 0.26, *P* = .04) and repair quality rankings at 12 months (*r*_*s*_ = 0.36, *P* = .02).

**Conclusions:**

These data support the finding that machines (SWEUS) are better at assessing torn rotator cuff tendon quality and whether that tendon will heal after repair than the ‘person’ performing the surgery. Supraspinatus tendons lose stiffness as they get older and when the tear is larger, likely explaining why retear post-cuff repair is more common with advanced age and larger tears.

Rotator cuff tears represent the most common musculoskeletal shoulder injury and comprise around half of all shoulder-related patient complaints.^1^^,^^24^^,^^25^^,^^29^ Rotator cuff tears occur most commonly in the supraspinatus tendon and are closely associated with trauma and advanced age.^34^^,^^37^ A hypothesized pathophysiology for rotator cuff tear begins with prolonged load-bearing tension upon the supraspinatus associated with overuse. In turn, tendinopathy develops in the undersurface of the supraspinatus tendon posterior to the long head of biceps. This portion of supraspinatus bears the most tension for stabilizing the glenohumeral articulation while performing overhead activities. This tendinopathy may progress to a partial-thickness tear on the tendon’s undersurface with continued tension. These partial-thickness tears can in turn progress into small or large full-thickness tears, before potentially resulting in a rotator cuff tear arthropathy.^36^^,^^38^

Arthroscopic rotator cuff repair (RCR) is a surgical procedure to treat rotator cuff tears by reattaching the torn supraspinatus tendon with sutures and anchors to the footprint of the greater tuberosity of the humerus.^10^ However, repair construct failures and failures of tendon-to-bone healing are relatively common complications of RCR,^22^ with 11%-57% of arthroscopic repairs reported to fail within 12 months^3^^,^^21^ and the majority failing within the first 6 months.^4^^,^^6^^,^^7^^,^^23^^,^^32^ Evaluation during revision surgery indicates that the major mechanism of RCR failure is by sutures pulling through the repaired tendon edge^8^ prior to healing at the enthesis. We hypothesize that following arthroscopic RCR, tendons with lower stiffness, referring to a measure of the tendon’s ability to counteract displacement with the application of stress,^11^ are more likely to retear by sutures pulling through the softer tendon at the enthesis prior to healing.^8^

Traditionally, surgeons have made intraoperative qualitative assessments of tissue quality, repair quality, and tendon mobility using a four-point Likert scale (detailed in Table I).^21^ However, the ability of these intraoperative assessments to predict repaired supraspinatus tendon integrity is not well understood.^18^ Another potential method to investigate the quality of a healing supraspinatus tendon is via shear wave elastography ultrasound (SWEUS), a recent advancement in ultrasound technology that enables an ultrasound machine to quantify the stiffness of soft tissues including rotator cuff tendons.^33^ SWEUS, as an application of ultrasound, is a method of measuring tendon stiffness that provides a quantitative score for stiffness at each point along the tendon. SWEUS quantifies the stiffness of a tendon by assessing how the cuff tendon deforms in response to stress.^5^

**Table I:** Intraoperative surgeon-grading scoring system^21^^,^^35^

	Fair (1 point)	Good (2 points)	Very good (3 points)	Excellent (4 points)
Quality of the Tendon	Thin, friable, does not hold suture	Patchy thickness, holds suture	Normal thickness, holds suture incidentally well	Thick and robust, holds suture very well
Tendon Mobility	Immobile and retracted	Poor mobility, can be barely pulled to the landing site	Mobile, can be pulled easily to the landing site	Mobile, can be pulled easily to the landing site
Repair Quality	Very weak repair	Repair not optimal	Relatively strong repair	Very strong repair

Tendons that are healthy and stiffer have higher conduction velocity values (approaching 10 m/s), appearing red in Figure 1. In blue are tendinopathic tendons with a lower velocity (approaching 0.05 m/s), where the tissue is softer and stiffness is reduced^20^^,^^33^ (Fig. 1). Studies by Taljanavic et al and Hou et al have noted normal, healthy male supraspinatus tendons to have an 8-9 m/s SWEUS velocity, while tendinopathic tendons, being those with reduced tendon integrity, have a SWEUS velocity of 5-7 m/s.^15^^,^^33^

**Figure 1:**
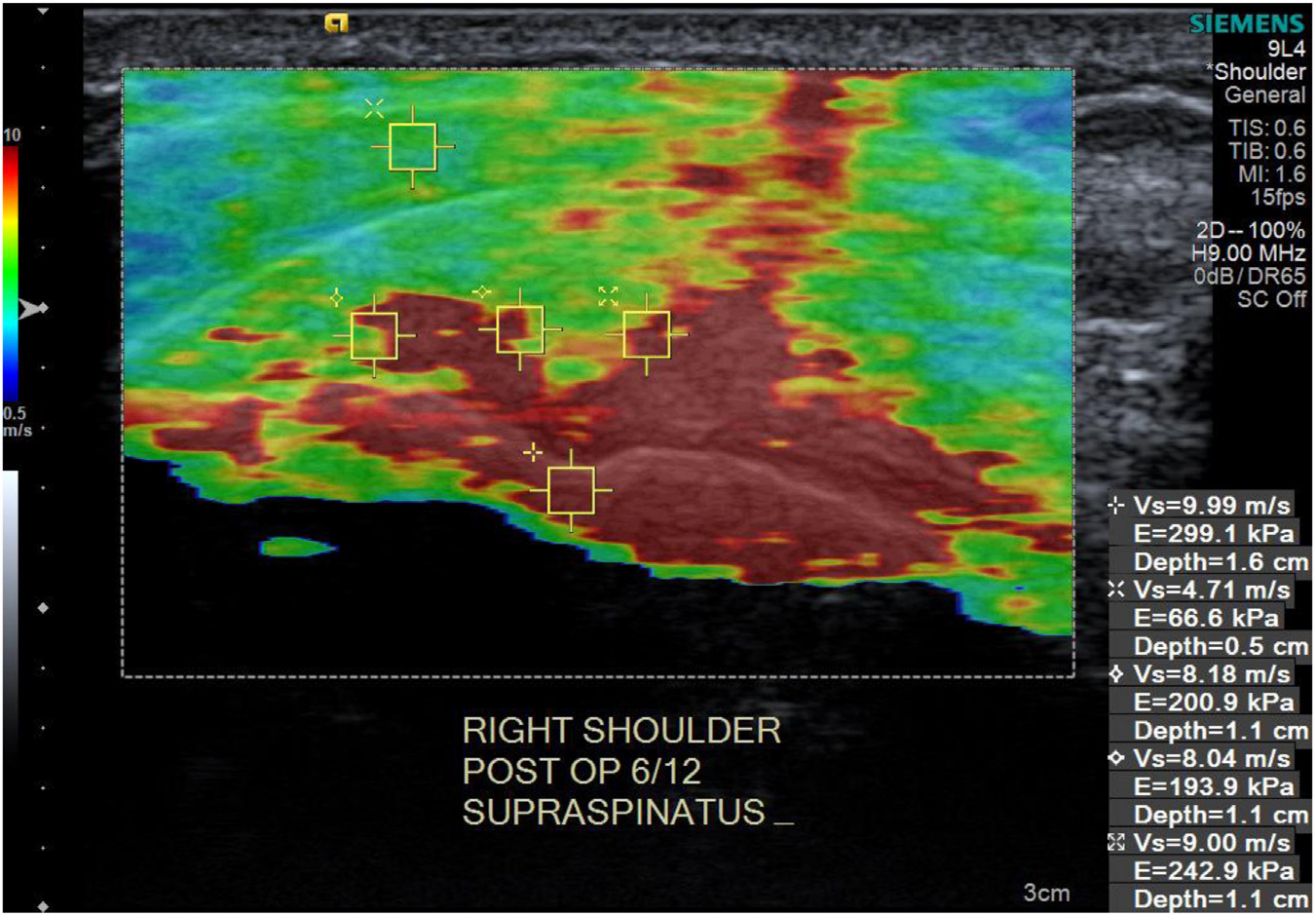
Shear wave elastography ultrasound ‘colour elastogram’ of the right supraspinatus tendon, where stiff tissue appears red and soft tissue appears blue from Hackett et al.^27^

To our knowledge, there has been no comparison of surgeon-ranked measures of tendon mobility, tendon quality, or repair quality against SWEUS velocity. The aims of this study, therefore, were to determine (1) How closely does a surgeon’s intraoperative ranking of tissue quality, repair quality, and tendon mobility correlate to supraspinatus tendon stiffness measured by SWEUS? (2) In the event of a correlation existing, at which postoperative time point are surgeon rankings most closely correlated with SWEUS? (3) Do intraoperative surgeon tendon mobility, tissue quality, and repair quality rankings and/or SWEUS velocity measurements of the supraspinatus tendon have an association with advanced age? (4) Do intraoperative surgeon tendon mobility, tissue quality, and repair quality rankings and/or SWEUS velocity measurements have an association with increased tear size? and (5) How do SWEUS velocity measurements of the supraspinatus tendon change up to 12 months postoperatively?

## Materials and methods

### Study design

This was a prospective case series evaluating the association between intraoperatively surgeon-ranked scores of tendon mobility, tissue quality, and repair quality and supraspinatus tendon stiffness postrepair.

### Ethics approval

Ethics approval for this project has been granted (SESLHD HREC Ethic: 2019/ETH09238).

### Cohort study design

#### Inclusion criteria

Inclusion criteria for this study were patients aged at least 18 years who underwent a primary arthroscopic-rotator cuff repair on a torn supraspinatus tendon by the same surgeon (G.M.) and returned for their follow-up 8-day, 6-week, 12-week, 6-month, and 12-month postoperative appointments, where SWEUS was conducted on three points of the repaired supraspinatus tendon by the same experienced musculoskeletal sonographer (L.H.).

#### Exclusion criteria

Exclusion criteria for this study were patients undergoing revision surgery, any repairs that involved concurrent shoulder procedures such as synthetic patches, calcific tendonitis débridement, distal clavicular resection or stabilization, partial repairs, significant muscle atrophy determined by B-mode ultrasound, glenohumeral arthritis (≥grade II), or patients with an avulsed fracture or isolated subscapularis tear. Furthermore, any patients whose tendons retore post-RCR were excluded from further statistical analyses once the tear was identified.

### Patient recruitment

Patients undergoing primary arthroscopic RCR by the same experienced shoulder surgeon (G.M.) upon being diagnosed with a supraspinatus tear were offered to participate in our study at their first appointment. During this preoperative appointment, patient data were collected, including patient age, sex, and whether their injury was caused by trauma via a standard questionnaire. At each preoperative appointment, a B-mode ultrasound was used to diagnose rotator cuff tears and also determine the tear size area (cm^2^) by the same experienced musculoskeletal sonographer. Following RCR, follow-up appointments were scheduled for 8 days, 6 weeks, 12 weeks, 6 months, and 12 months postoperatively, with both SWEUS and traditional B-mode ultrasound to ensure the repair remained intact. At each of these appointments, SWEUS velocity measurements were taken at three set points (labelled ‘medial’, ‘mediolateral’, ‘lateral’) along the supraspinatus tendon to measure stiffness, described below.

### Surgical arthroscopic rotator cuff repair technique

Indications for RCR were patient pain, full thickness tears, or partial thickness tears more than 50% thickness that were also deemed repairable on ultrasound. For all patients, arthroscopic RCR employing the undersurface technique approach was used, detailed elsewhere.^31^ An interscalene block was administered to each patient on the operative side, before placing them in the beach-chair position when they were then prepped and draped. A posterior portal was created to insert the arthroscope, while a spinal needle was used to create the lateral portal. Then, using scalpel partial thickness tears were converted to full-thickness tears with an 11-blade scalpel before the humeral greater tuberosity was débrided. Then, Opus Magnum Sutures (Smith and Nephew, North Ryde, NSW, Australia) were used to repair the torn tendon through the knotless suture technique.

Using an Opus Smart-Stitch Suture Devie (Smith and Nephew, North Ryde, NSW, Australia), the torn supraspinatus tendon was held in position before a Number 2 polyester inverted mattress suture was inserted via the lateral portal into the rotator cuff. The bone of the landing site had a hole punched through it via the lateral portal before an Opus Magnum Knotless Implant (Smith and Nephew, North Ryde, NSW, Australia) had both limbs of the suture passed through it for insertion and deployment into the bone hole. Then, the suture was wound through the anchor, enabling the tendon to be reduced down to the humeral head, before being locked into the anchor. Postoperatively, the patient was fastened into an abduction sling with a small cushioning pillow to be worn daily for the first 6 weeks (DonJoy, Frenchs Forest, NSW, Australia).

### Intraoperative surgeon ranking method

During surgery, the tissue quality, repair quality, and tendon mobility gradings were assessed and recorded by the same surgical team. Each of the three quality rankings was scored on a 4-point Likert scale (Fair, good, very good, or excellent), using the criteria in Table I. The operative duration was recorded as the time taken between glenohumeral joint visualization (via the posterior portal) and skin closure commencement.

### Shear wave elastography ultrasound examination

Following arthroscopic RCR, at 8 days, 6 weeks, 12 weeks, 6 months, and 1 year, a shear wave elastography ultrasound assessment of each patient’s supraspinatus tendon was taken. All SWEUS velocity values were obtained through measurements taken by a single experienced musculoskeletal sonographer (L.H.) using the Linear 9L4 transducer of a ‘Siemens Acuson S3000’ ultrasound machine (Siemens Medical Solutions, Malvern, PA, USA). Both our sonographer (L.H.) and ultrasound system employed in this study have been shown to be very reliable in evaluating the stiffness of supraspinatus tendons with SWEUS.^14^ The SWEUS velocity values obtained with our ultrasound machine ranged between 0.05 m/s and 10 m/s.

All SWEUS measurements were taken with patients seated and the shoulder in neutral rotation with zero degrees of abduction. The sonographer faced each patient anteriorly. Patient elbows were bent to 90^°^ with the hand supinated and resting in their lap. Each patient’s arm was extended 35^°^ from neutral. The greater tuberosity of the humerus and acromion, as lateral and medial landmarks, respectively, served as reference points for ultrasound transducer positioning. The transducer was positioned posteriorly in the supraspinatus fossa, enabling the supraspinatus tendon to be longitudinally visualized, resembling a “birds-beak”. Elastography stiffness measurements of the supraspinatus tendon were taken with a view of the anterior supraspinatus tendon, which was best viewed 2 mm posterior to the biceps at the tendon footprint-articular cartilage junction of the humerus.

SWEUS velocity measurements of stiffness were taken at three points along the tendon and at two controls. The two control tissues were selected to give contrasting velocity values, one in the deltoid muscle (low velocity, soft) superior to the supraspinatus tendon as a ‘negative control’ and another in the bone of the humeral head (high velocity, stiff) as a ‘positive control’.

**Figure 2:**
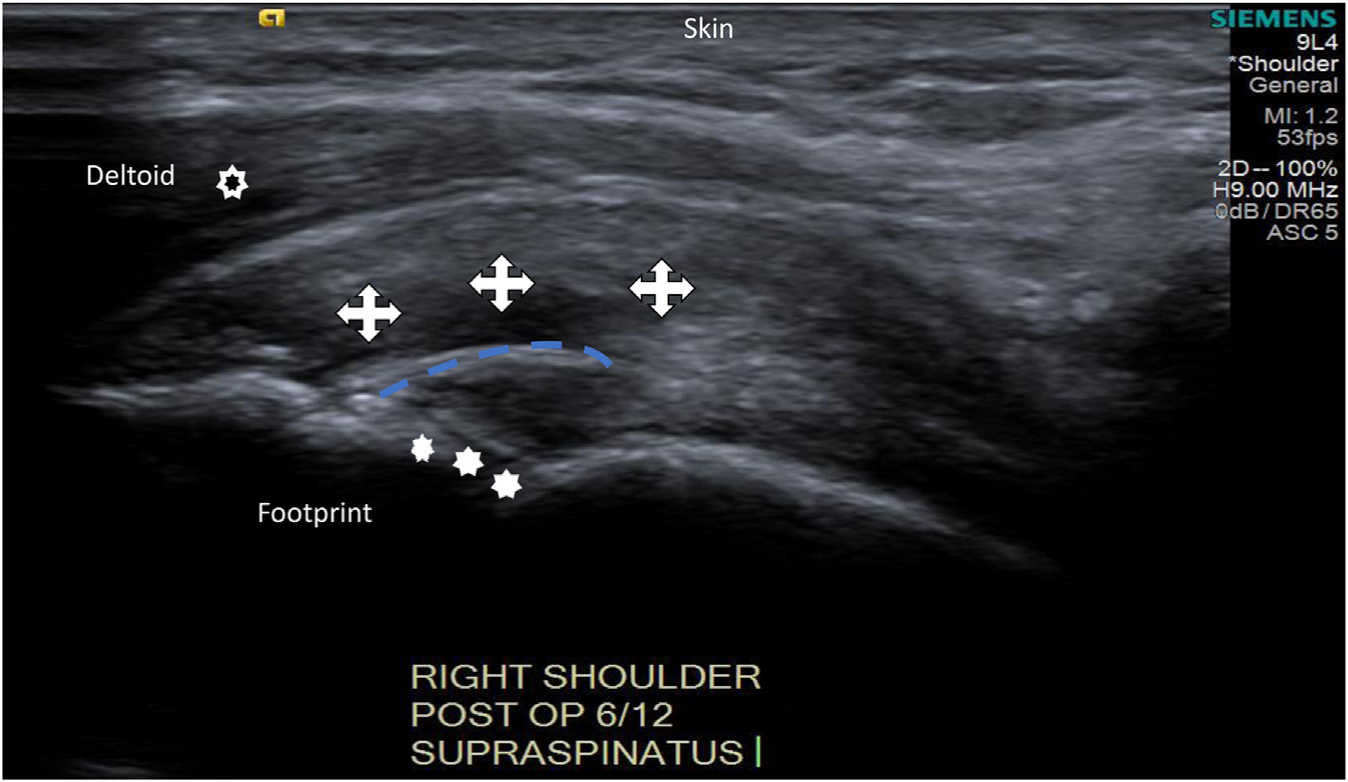
Ultrasound image showing three distinct supraspinatus tendon location (crosses), humeral head (stars), and deltoid (hollow circle) measurements 3 mm apart.

Healing supraspinatus tendon stiffness measurements were taken at three points along the supraspinatus, with SWEUS velocity values recorded at these three locations along the tendon-bone interface. Measured regions were taken by placing the sample box at the insertion site of the supraspinatus tendon into the greater tuberosity laterally (labelled the ‘Lateral point’, at the tendon-bone interface), 3 mm medially to the tendon-bone interface (labelled the ‘Mediolateral point’), and 6 mm medially to the tendon-bone interface (labelled the ‘Medial point’) (Fig. 2). Orthogonal lines are depicted to account for supraspinatus tendon curvature.

SWEUS velocity values were generated as a measure of stiffness and digitally recorded for each patient. These areas of interest along the supraspinatus tendon were chosen to avoid bias caused by suture material upon the stiffness of the measured region.

### Statistical analysis

All data analysis was performed using IBM SPSS Statistics 26 for Windows (IBM Corp., Armonk, NY, USA), with significance set at *P* < .05. A one-way analysis of variance (ANOVA) with repeated measures was used to assess the tendon stiffness changes via SWEUS velocity measurements at each of the three points along the supraspinatus tendon.

Spearman’s correlation coefficient was generated to examine the strength of correlation between SWEUS velocity values obtained at each point along the supraspinatus tendon for each time point and each of the intraoperatively surgeon-ranked tissue quality, tendon mobility, and repair quality scores. Similarly, Spearman’s rank correlation coefficients were employed to determine the correlation of surgeon intraoperative rankings to age and tear size. Pearson’s correlation coefficient was used for parametric data to evaluate if age and tear size were significantly correlated to the SWEUS velocity of the healing supraspinatus tendon postrepair.

Repeated measures three-way ANOVAs were used to determine if there were significant differences in the correlation of SWEUS velocity to age and tear size at each of the three sites along the tendon across each postoperative time point.

## Results

### Participant demographics

Fifty patients were recruited for this study. SWEUS velocity scores were recorded at 8 days, 6 weeks, 12 weeks, 6 months, and 12 months. Two patients did not attend follow-up appointments leaving a cohort of 48 patients, of which 28 were male and 20 were female. Ages ranged from 24 to 85 years with a median age of 57 years. Six patients retore their tendons: three at 6 weeks, two at 12 weeks, and one at 6 months.

### Control measurements

The mean (±SD) SWEUS velocity of the humeral head (as the positive control) was unchanged from 8 days (9.78 ± 0.2 m/s) to 12 months (9.86 ± 0.1 m/s). The deltoid muscle (as the negative control) increased in SWEUS velocity from 3.4 ± 0.9 m/s at 8 days to 4.1 ± 1.0 m/s at 12 months post-RCR (*P* = .03).

### Changes in the SWEUS velocity of the repaired tendon

**Figure 3:**
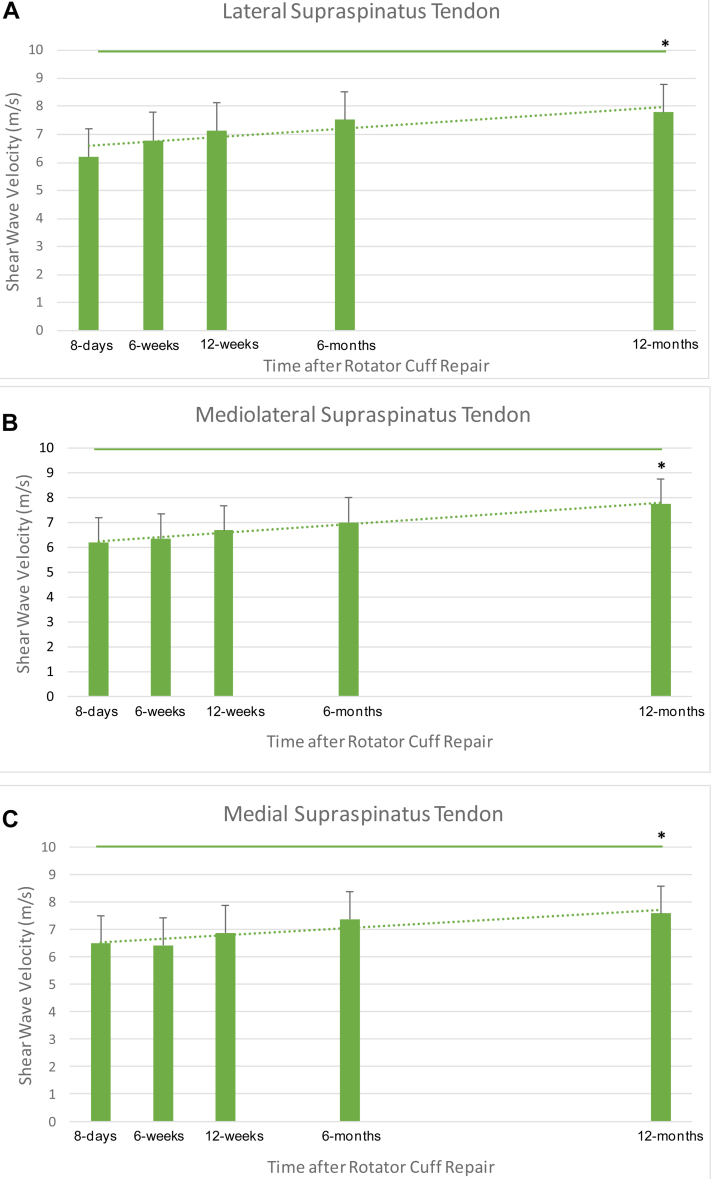
Shear wave velocity (±SD) measurements of the healing supraspinatus tendon at the (**A**) lateral (**B**) mediolateral (3 mm medial), (**C**) medial (6 mm medial) points of the supraspinatus tendon along its insertion site post arthroscopic rotator cuff; n = 42, ∗*P* < .05 using one-way ANOVA. Dotted line denotes linear trendline. *ANOVA*, analysis of variance.

The SWEUS stiffness of the repaired supraspinatus tendon was found to improve in all three measured locations with time following RCR, with an average overall increase in elastography stiffness of 22% from 8 days to 12 months (*P* = .0001) (Fig. 3).

At the lateral point of the arthroscopically repaired supraspinatus tendon, SWEUS velocity increased from 6.2 ± 0.2 m/s at 8 days to 7.8 ± 0.3 m/s at 12 months postrepair (*P* = .0001). At the mediolateral point, SWEUS velocity increased from 6.2 ± 0.2 m/s at 8 days to 7.8 ± 0.3 m/s at 12 months postrepair (*P* = .001). Medially, SWEUS velocity increased from 6.5 ± 0.2 m/s at 8 days to 7.6 ± 0.3 m/s at 12 months postrepair (*P* = .03). There were significant differences in the SWEUS-measured stiffness of the repaired supraspinatus tendon at all three measured locations (*P* = .0001) across all postoperative time points (*P* = .0001), confirmed by three-way ANOVA.

### Tendon quality rankings vs. SWEUS velocity

The surgical team ranked the quality of the torn edge of the supraspinatus tendon as very poor for one patient (2%), poor for 8 patients (16%), very good for 11 patients (22%), and excellent for 31 patients (62%) intraoperatively. Intraoperative surgeon-ranked measures of tendon quality were weakly associated (*r*_*s*_ = 0.02−0.27, *P* = .045−.45) with SWEUS velocity scores of the repaired tendon at all measured points along the supraspinatus tendon insertion site from 8 days to 12 months postrepair.

**Figure 4:**
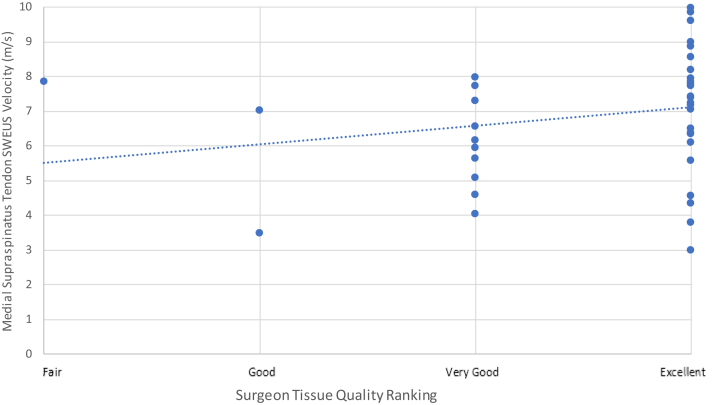
Surgeon-ranked tendon quality scores vs. SWEUS velocity stiffness of the medial supraspinatus tendon (6 mm medial to the tendon-bone interface) at 12-weeks post repair using Spearman’s rank correlation analysis *n* = 42. Dotted line denotes linear trendline. *SWEUS*, sheer wave elastography ultrasound.

The most consistent association of SWEUS velocity to surgical team tendon quality rankings was at the medial point along the supraspinatus tendon (6 mm medial to its insertion), reaching statistical significance at 12 weeks postoperatively (*r*_*s*_ = 0.27, *P* = .045) (Fig. 4).

### Tendon mobility rankings vs. SWEUS velocity

Surgical team rankings of intraoperative supraspinatus tendon mobility were relatively granular: excellent for 47 patients (94%), very good for 2 patients (4%), and very poor for 1 patient (2%). Intraoperative surgeon-ranked measures of tendon mobility correlated weakly to SWEUS velocity scores across all measured points along the supraspinatus tendon from 8 days to 12 months postrepair *r*_*s*_ = −0.04−0.26, *P* = .045−.47 using Spearman’s rank correlation coefficient.

**Figure 5:**
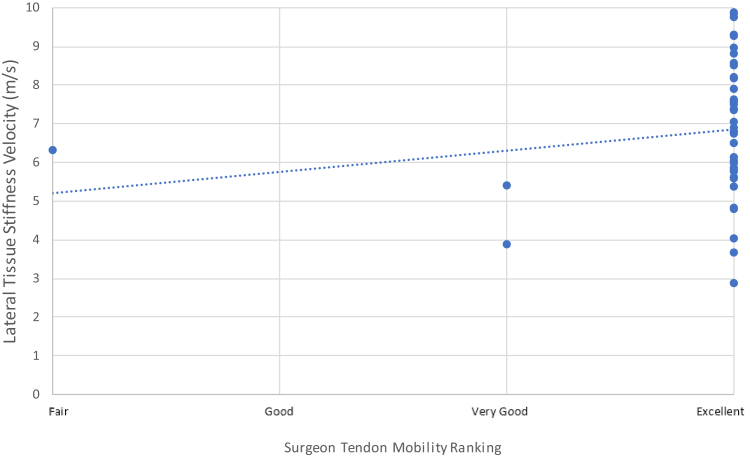
Surgeon-ranked tendon mobility scores vs. lateral shear wave ultrasound velocity at 6 weeks postrotator cuff repair using Spearman’s rank correlation *n* = 44. Dotted line denotes linear trendline.

At the lateral point along the supraspinatus tendon, there was a significant correlation between surgeon-ranked tendon mobility scores and SWEUS velocity measurements 6 weeks postoperatively (*r*_*s*_ = 0.26, *P* = .046) (Fig. 5).

### Repair quality rankings vs. SWEUS velocity

The surgical team graded the repair quality of the supraspinatus tendon as excellent for 46 patients (92%), very good for 3 patients (6%), and very poor for 1 patient (2%). Intraoperative surgeon-ranked measures of the repair quality correlated weakly with SWEUS velocity measurements along all three measured points of the supraspinatus tendon for all postoperative time points (*r*_*s*_ = −0.1 to 0.36, *P* = .017-.40).

**Figure 6:**
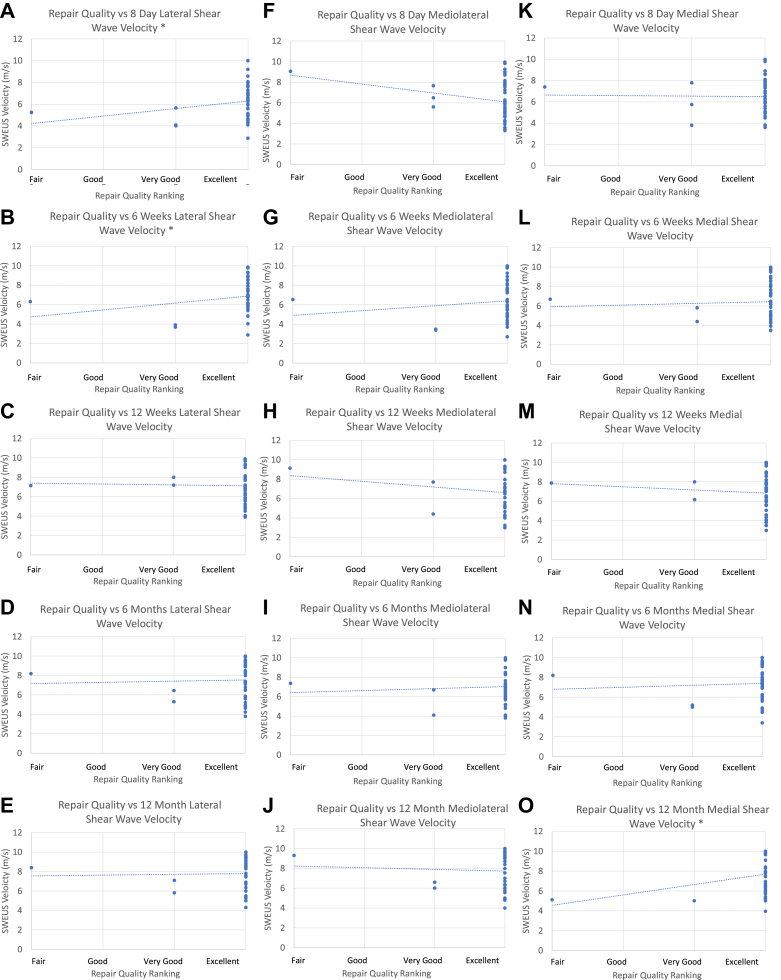
Surgeon ranked repair quality scores vs. (**A-E**) lateral, (**F**-**J**) mediolateral, and (**K-O**) medial shear wave velocity values at 8 days, 6 weeks, 12 weeks, 6 months, and 12 months postrotator cuff repair. ∗Statistical significance was reached at the lateral point of the supraspinatus tendon at 8 days (*r*_*s*_ = 0.32, *P* = .014) and 6 weeks (*r*_*s*_ = 0.30, *P* = .024), and at the medial point at 12 months (*r*_*s*_ = 0.36, *P = .017*), ∗*P* < .05, *n* = 42 using Spearman’s rank correlation coefficient. Dotted line denotes linear trendline.

At the lateral point along the supraspinatus tendon, there was a significant positive correlation between repair quality rankings and SWEUS velocity at 8 days (*r*_*s*_ = 0.32, *P* = .014) and 6 weeks (*r*_*s*_ = 0.30, *P* = .024) postoperatively (Fig. 6). For the mediolateral point along the supraspinatus tendon (3 mm medial to the tendon-bone interface), there was no significant correlation (*P* < .05) for any given postoperative time point. At the medial point (6 mm medial to the tendon-bone interface) along the supraspinatus tendon, there was a significant positive correlation (*r*_*s*_ = 0.36, *P* = .017) at 12 months postoperatively.

## Age vs. intraoperative surgeon rankings

### Age vs. surgeon tissue quality ranking

**Figure 7:**
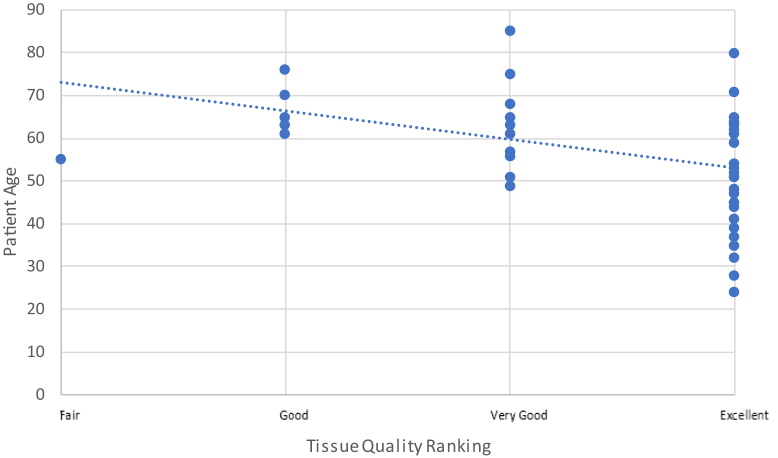
Surgeon-ranked tissue quality scores vs. patient age at the time of rotator cuff repair using Pearson’s correlation coefficient *n* = 48. Dotted line denotes linear trendline.

The surgical team ranked the quality of the tissue of the torn edge of the supraspinatus, at the time of repair, to be less robust in older patients as compared to younger patients (*r* = −0.49, *P* = .0001) (Fig. 7).

### Age vs. surgeon tendon mobility and repair quality rankings

In contrast to tissue quality rankings, we found no association between intraoperatively surgeon-ranked tendon mobility scores and patient age (*r* = −0.0127, *P* = .465). Similarly, there was no association between intraoperative repair quality surgical team rankings and patient age (*r* = −0.191, *P* = .09).

## Age vs. SWEUS velocity

**Figure 8:**
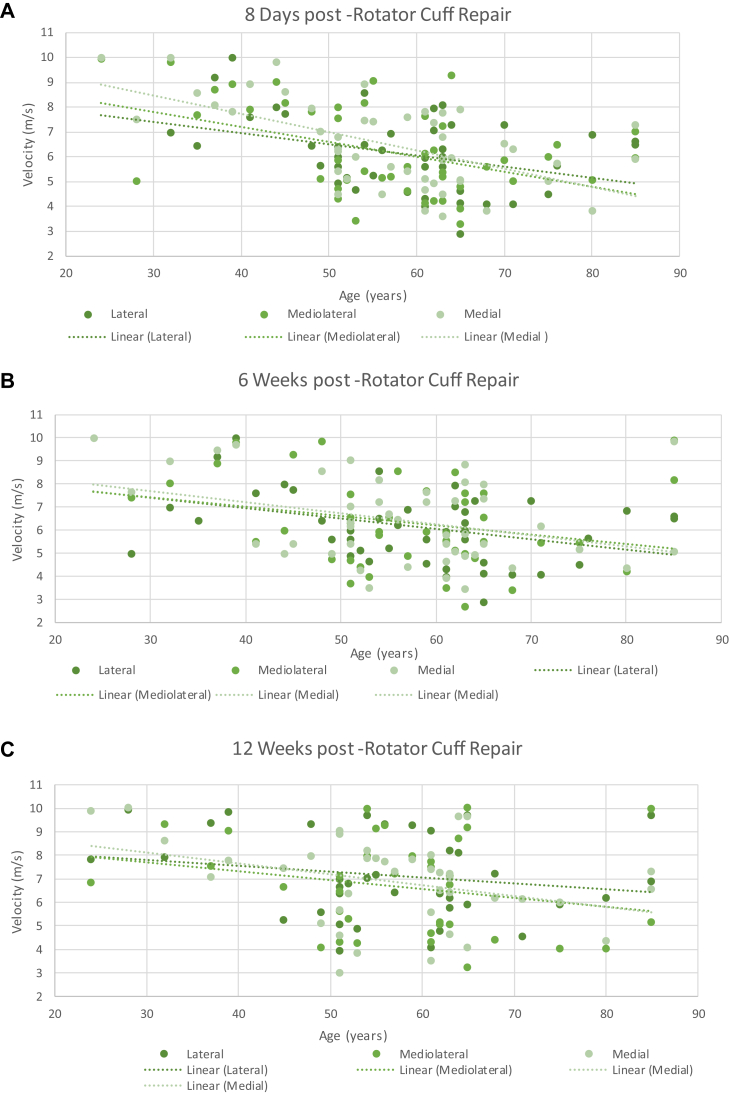

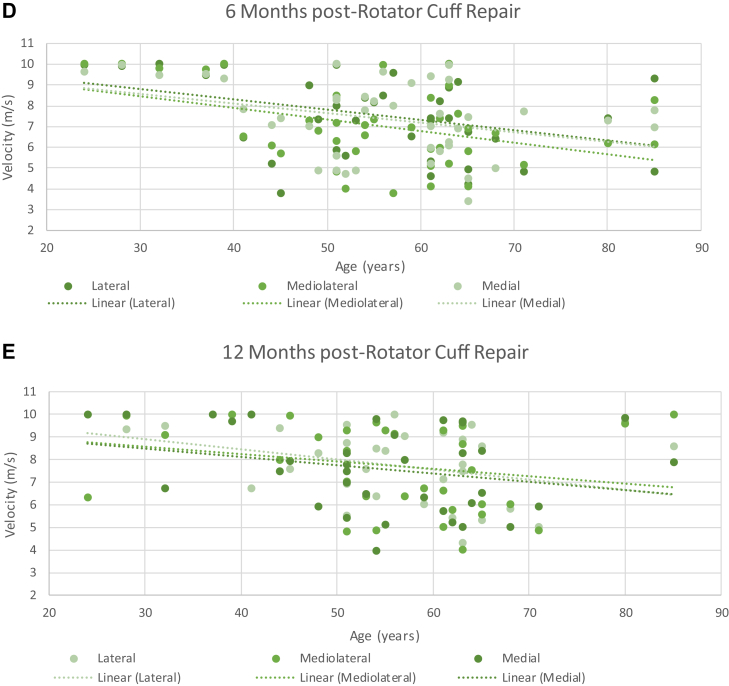
The association of age and SWEUS velocity at the lateral, mediolateral, and medial points along the supraspinatus tendon insertion (**A**) 8 days, (**B**) 6 weeks, (**C**) 12 weeks, (**D**) 6 months, and (**E**) 12 months post-RCR *n* = 42. Dotted line denotes linear trendline. There were significant differences in SWEUS velocity between patients of different ages (*P* = .0001) across every postoperative time point (*P* = .0001), significant at all three measured tendon locations (*P* = .01) using three-way ANOVA analysis. *SWEUS*, sheer wave elastography ultrasound; *RCR*, rotator cuff repair; *ANOVA*, analysis of variance.

Advanced age was associated with lower SWEUS-measured supraspinatus tendon stiffness postrepair, consistent at each time point (*P* = .0001) and significant at all three measured tendon locations (*P* = .0001) using three-way ANOVA analysis. Age was negatively associated with supraspinatus tendon stiffness across every time point post-RCR and for all three tendon locations, but most obvious at the medial point along the torn tendon edge at 8 days postrepair (*r* = −0.58, *P* = .00001) (Fig. 8).

## Tear size vs. intraoperative surgeon rankings

### Tear size area vs. tissue quality ranking

**Figure 9:**
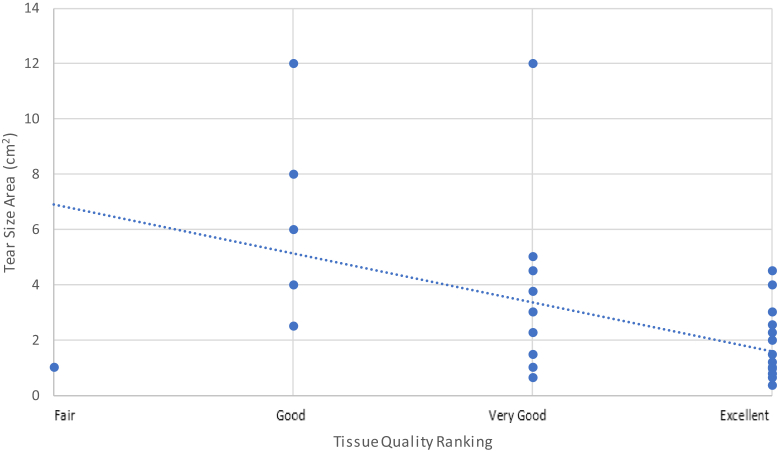
Surgeon-ranked tissue quality scores vs. tear size area at the time of rotator cuff repair using Pearson’s correlation coefficient, *n* = 48. Dotted line denotes linear trendline.

The surgical team ranked the quality of the tissue of the torn edge of the supraspinatus tendon, at the time of repair, to be less robust in patients with larger tear size areas as compared to smaller tears (*r*_*s*_ = −0.565, *P* = .000014) (Fig. 9).

### Tear size area vs. tendon mobility and repair quality rankings

In contrast to tissue quality rankings, we found no association between supraspinatus tear size area and intraoperatively ranked tendon mobility scores (*r*_*s*_ = −0.14, *P* = .159 nor intraoperative surgeon repair quality rankings (*r*_*s*_ = −0.187, *P* = .101.

## Tear size vs. SWEUS velocity

**Figure 10:**
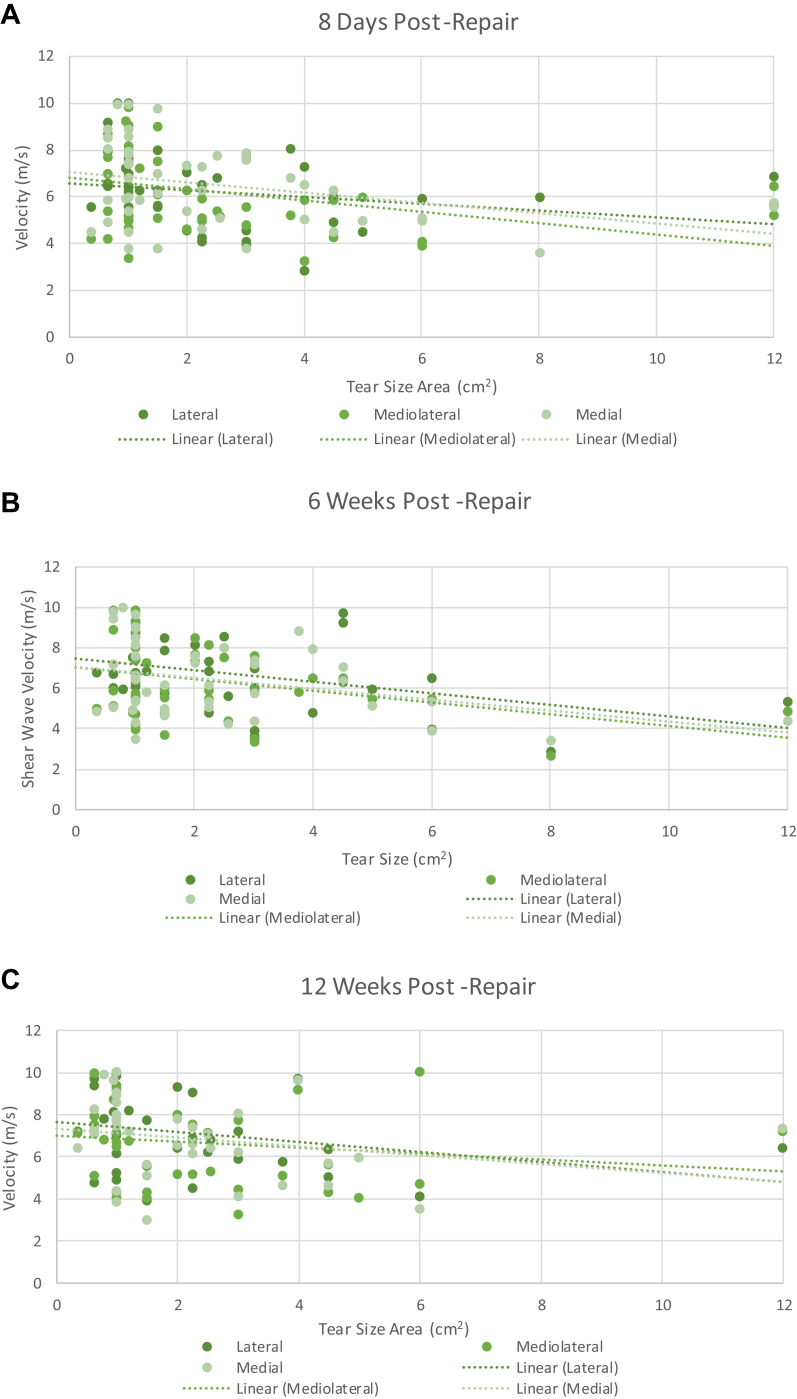

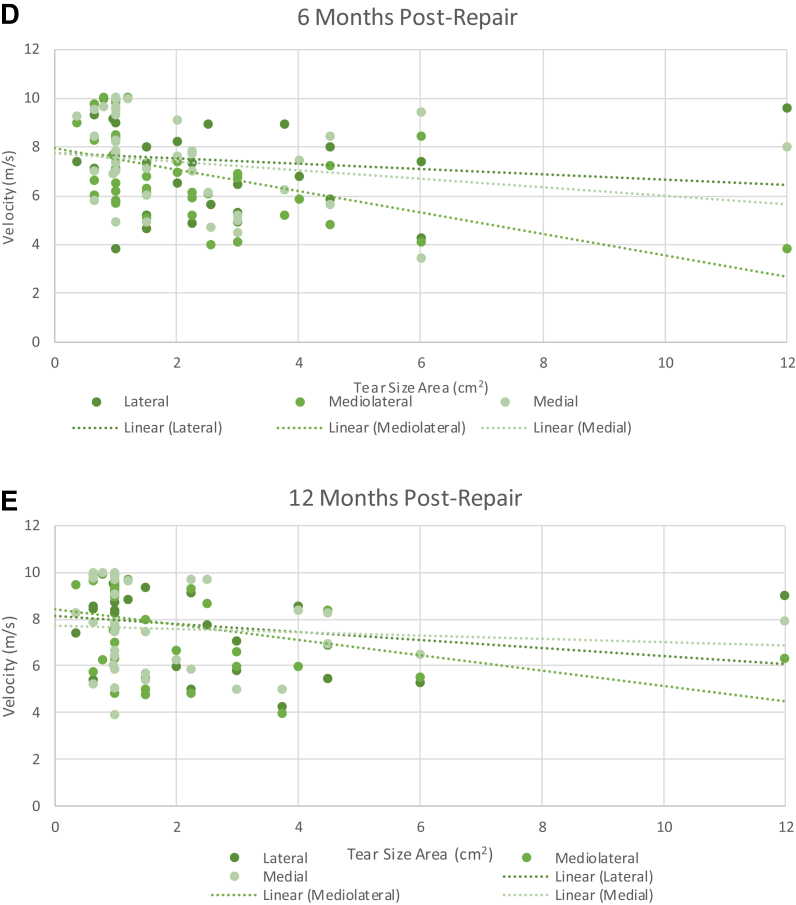
The association of tear size area and SWEUS velocity at lateral, mediolateral, and medial points along the supraspinatus tendon insertion was measured at (**A**) 8 days, (**B**) 6 weeks, (**C**) 12 weeks, (**D**) 6 months, and (**E**) 12 months post-RCR *n* = 46. Dotted line denotes linear trendline. There were significant differences in SWEUS velocity between tendons with different tear size areas (*P* = .0001) across every postoperative time point (*P* = .034) and tendon location (*P* = .0001) using three-way ANOVA analysis. *SWEUS*, sheer wave elastography ultrasound; *RCR*, rotator cuff repair; *ANOVA*, analysis of variance.

The average tear size area among the cohort was 2.6 cm^2^, ranging from 0.4-12.0 cm^2^. Larger rotator cuff tear size areas were associated with lower supraspinatus tendon stiffness as measured by SWEUS velocity postoperatively. There were significant differences in the stiffness of repaired supraspinatus tendons with different tear size areas (cm^2^) (*P* = .0001 using 3-way ANOVA analysis), along with significant differences in SWEUS velocity between different follow-up time points (*P* = .034). The negative association between tear size area and SWEUS measured stiffness was consistent at each time point post-RCR at all three tendon locations but most obvious in the mediolateral tendon at 6 months postrepair (*r* = −0.50, *P* = .02) (Fig. 10).

## Discussion

The major findings of this study were that SWEUS velocity measurements were better at assessing differences in tissue quality following arthroscopic RCR of the supraspinatus tendon than qualitative intraoperative rankings made by the surgical team. The correlations between SWEUS-determined tendon stiffness and surgeon tissue quality, tendon mobility, and repair quality rankings were modest at best. The supraspinatus tendon became 22% stiffer, as determined by SWEUS velocity, at the repaired insertion site from 8 days to 12 months post-RCR, and was observed to become stiffer laterally earlier than it did medially. Tendons in older patients and those with larger tears were found to have reduced supraspinatus tendon stiffness as measured by SWEUS after RCR.

SWEUS has been shown to be a reliable tool for diagnosing tendinopathy of the rotator cuff.^17^ Hackett et al,^14^ at our institution, examined SWEUS’ reliability in assessing supraspinatus tendons in 10 normal and 10 tendinopathic participants using virtual touch imaging quantification of the Siemens ACUSON S3000 ultrasound system. Hackett et al^14^ found SWEUS velocity measurements to exhibit excellent intraobserver (ICC = 0.96) and moderate interobserver reliability (ICC = 0.45), similar to study findings by Baumer et al^2^ (ICC = 0.87). Hou et al^15^ also evaluated the association of SWEUS velocity to the degree of supraspinatus tendinopathy, finding the morphological grade of supraspinatus tendinopathy on B-mode ultrasound to be associated with decreased supraspinatus tendon stiffness (*P* < .003), demonstrating shear wave elastography’s capacity to identify tendons with decreased tensile properties.

We observed increasing SWEUS velocity in all three measured points of the repaired supraspinatus tendon from 8 days to 12 months, reflecting improved material properties of the tendon. The improved SWEUS velocity of the healing supraspinatus tendon may result from repair-phase tendon remodeling. Yoo et al found, in a study of 65 RCRs, a complete restoration of normal fibrillar pattern on ultrasound in 85% of repaired tendons at 6 months, reduced surface irregularity in 45% and a loss of hypoechoic texture in 70%. Thus, our data support the hypothesis that the mechanical stiffness of the rotator cuff tendon is increased with an improved fibrillar pattern, surface regularity, and tendon texture. The observed increase in stiffness of repaired supraspinatus tendons in our study is also consistent with the findings of a study in 60 rats by Galatz et al, where up to 56 days after RCR, a 66% improvement in supraspinatus tendon stiffness was observed when testing the tendon load-to-failure. There have been several other studies in humans using SWEUS to assess tendon healing. Itoigawa et al^26^ evaluated 60 patients from one week to 6 months post-RCR and found decreasing stiffness of the supraspinatus tendon at the anteromedial anchor following double row repair, albeit with a different ultrasound machine (Aixplorer System; SuperSonic Imagine, Aix-en-Provence, France) and without reporting the decreased velocity values. Nocera et al^26^ used SWEUS to evaluate the postrepair stiffness of supraspinatus tendons in 12 patients, finding an initial decrease in stiffness at 3 months (*r* = −0.73, *P* = .005), and noting a trend of improved stiffness at 6 months postoperatively.

Huijsmans et al^16^ reported that tendons with poorer surgeon-ranked quality had an increased likelihood of rotator cuff retear in 242 patients undergoing RCR. Similarly, Cummins and Murrell at our institution showed that in a cohort of 342 consecutively repaired rotator cuffs with 21 failures (defined as “suture pull-through at the tendon-suture interface”), lower tendon quality was associated with failure, with quality scores significantly lower at revision surgery compared with primary surgery.^8^ Intraoperative surgeon-ranked tissue quality scores, however, were weakly associated with postoperative tendon stiffness in our present study and only reached significance at the medial point along the tendon at only one postoperative interval (12 weeks). Therefore, our data suggest that SWEUS is a more sensitive method of assessing tissue quality than qualitative intraoperative surgical team rankings.

A study by Kim et al^18^ in 2016 used an intraoperative tensiometer to measure the tension of the repaired rotator cuff tendon during arthroscopic RCR in 132 patients with full-thickness tears and found an inverse correlation between the tension of the repaired tendon (as a measure of mobility) and postoperative supraspinatus tendon integrity determined through magnetic resonance imaging. Kim et al reported no association between qualitative surgeon-ranked scores for tendon quality and repaired supraspinatus tendon integrity. The poor correlations between surgeon-ranked tendon quality or mobility and postoperative supraspinatus stiffness suggest these intraoperative surgeon-ranked factors are of little use in predicting postoperative tendon integrity.

Studies involving 447,^30^ 1600,^9^ and 1000^21^ patients in multiple regression analyses have shown advanced age to be associated with an increased risk of retear/nonhealing following supraspinatus tendon repair. In our study, older age was associated with reduced supraspinatus tendon stiffness as assessed by both surgeon rankings of tissue quality (*r*_*s*_ = −0.49, *P* = .0001) and SWEUS (*r* = −0.55, *P* = .00002) post-RCR. Fontenelle et al^12^ also found higher supraspinatus tendon stiffness as assessed by SWEUS in 20-35-year-olds with uninjured tendons, as compared to those aged more than 60 years, who are at high risk of degenerative changes. Plate et al^27^ reported 28 older rats to have significantly poorer collagen organization and worse fibroblastic malalignment than 28 younger rats at the tendon-bone junction 8 weeks following RCR. Thus, the reduced tendon stiffness we observed in older patients is likely to be indicative of tendon quality worsening with advanced age.

Our study also found that larger tears were associated with reduced postoperative SWEUS-measured supraspinatus tendon stiffness (*r* = −0.55, *P* = .000008) and poorer intraoperative surgeon rankings of tissue quality (*r*_*s*_ = −0.57, *P* = .00001). Krepkin et al^19^ similarly found the SWEUS velocity of 9 repaired supraspinatus tendons to be negatively associated with tear size. A multiple logistic regression analysis in 1600 patients by Diebold et al found a significant association between larger initial tear sizes and increased rates of retear. Thus, we hypothesize that this decrease in supraspinatus tendon stiffness observed with larger tears may explain the greater rate of retear in those with larger initial tear size areas.^35^

Our study strengths included the use of a single, experienced musculoskeletal sonographer whose intrauser reliability has been shown to be high,^14^ who conducted all shear wave ultrasound assessments, while the senior author G.M. performed and graded each RCR. In addition, our study used bone for a positive control and muscle for a negative control and a standardized sample box window size which improved measurement reliability.^28^

There are some weaknesses of our study design which should be considered. A preoperative measurement of supraspinatus tendon stiffness would have more clinical relevance for presurgical prognosis. The use of a single surgeon, sonographer, and ultrasound machine reduced our study’s external validity and applicability to those using alternative repair, intraoperative ranking, and velocity measurement techniques.^13^

## Conclusion

The data from this study are consistent with the hypothesis that tendons lose stiffness (ie, have inferior material properties) as they get older and when the tear size area is larger. This reduction in tendon quality likely explains, in part at least, why age and tear size are associated with increased retear rates post-RCR. SWEUS also was able to show that the supraspinatus tendon, as it heals, becomes stiffer over time postrepair. Our study found the correlations between SWEUS velocity and surgeon tissue quality rankings were modest at best. Overall, machines (through SWEUS) were better at assessing torn rotator cuff tendon quality than the person performing the surgery.

## Disclaimers

Funding: No funding was disclosed by the authors.

Conflicts of interest: The authors, their immediate families, and any research foundations with which they are affiliated have not received any financial payments or other benefits from any commercial entity related to the subject of this article.
